# Geroscience approaches to increase healthspan and slow aging

**DOI:** 10.12688/f1000research.7583.1

**Published:** 2016-04-29

**Authors:** Simon Melov

**Affiliations:** 1 Buck Institute for Research on Aging, Novato, CA, USA

**Keywords:** Geroscience, healthspan, slow aging, lifespan, longevity

## Abstract

For decades, researchers in the biology of aging have focused on defining mechanisms that modulate aging by primarily studying a single metric, sometimes described as the “gold standard” lifespan. Increasingly, geroscience research is turning towards defining functional domains of aging such as the cardiovascular system, skeletal integrity, and metabolic health as being a more direct route to understand why tissues decline in function with age. Each model used in aging research has strengths and weaknesses, yet we know surprisingly little about how critical tissues decline in health with increasing age. Here I discuss popular model systems used in geroscience research and their utility as possible tools in preclinical studies in aging.

## Introduction

We are at a tipping point in the biology of aging—from lifespan extension *per se* to maintaining and extending health in late life. Since the early 1980’s, there have been serious efforts to use genetic approaches to extend lifespan in model systems such as *Caenorhabditis elegans* ^1– 6^, *Drosophila* ^7– 15^, and, increasingly, mice ^16, 17^. Collectively, such efforts fall under the catch-all term “geroscience”, which describes interdisciplinary efforts to better understand the biology of aging with a view towards improving healthcare in the elderly ^18^. Recently, the tried and true genetic approaches of the 1990’s and early 2000’s in geroscience research have been increasingly giving way to a plethora of pharmacological approaches to extend lifespan. This has been in conjunction with efforts to simultaneously increase healthspan ^19– 28^, thereby providing a preclinical rationale for similar studies in human beings.

It has been reported that lifespan and healthspan can be extended in invertebrates using a variety of pharmacological approaches, including single antioxidants through small molecule screens and natural compounds ^23^ as well as some anticonvulsants ^29^. Not to be outdone, there are also supporting data for lifespan/healthspan extension in mice using repurposed US Food and Drug Administration (FDA)-approved drugs, novel chemical compounds, and biologicals ( Table 1). Before examining key concepts in geroscience that drive a lot of the excitement in the pharmacology of lifespan/healthspan extension, it is necessary to first of all define what we mean by aging and healthspan. This is particularly germane in the model systems most commonly used in the biology of aging. By no means is the definition of such terms straightforward, and eminent figures in the field have spent considerable effort clarifying such apparently simple concepts. Caleb Finch of USC in his highly respected tome *Longevity, Senescence, and the Genome* ^30^ devoted several chapters towards defining what is meant by “aging”—or, as he prefers to denote it, “senescence”. More recently, similar efforts to define aging/senescence have been discussed at length by several other investigators ^31– 34^. Some popular definitions of aging in a geroscience context have included the following:

- use of mortality kinetics of an aging population to derive a mathematical definition
- the length of life after the reproductive period
- the probability of death with increasing age

For the purposes of this article, the term “aging” refers to post-reproductive changes that adversely affect lifespan. However, to define healthspan in the context of geroscience is perhaps even more difficult.

**Table 1.** Selected healthspan or lifespan studies using pharmacological interventions in geroscience research. Independent Replication refers to whether or not an independent group replicated the original reported result. X refers to failure to replicate, while a check mark indicates the key finding was replicated. Challenge Publication lists the reference where the independent group confirmed or failed to replicate key aspects of the initial report.

Reference	Year Published	Organism	Intervention	Lifespan/Healthspan Indication	Independent Replication	Challenge Publication
102	2000	*C. elegans*	EUK-8, EUK-134	Lifespan	X	103
104	2002	*Drosophila*	4-phenylbutyric acid	Lifespan	-	
29	2005	*C. elegans*	Anti-convulsants	Lifespan	-	
105	2006	*C. elegans*	Blueberry extract	Lifespan	-	
106	2007	*C. elegans*	Antidepressant	Lifespan	X	107
108	2008	*C. elegans*	Lithium	Lifespan	-	
109	2009	Mouse	Rapamycin	Lifespan	✓	110
111	2011	*C. elegans*	Amyloid-binding compounds	Lifespan	-	
112	2011	*Drosophila*	Pyrrolidine dithiocarbamate	Lifespan	-	
99	2013	Mice	Metformin	Lifespan/Healthspan	-	
91	2013	Mice	GDF11	Healthspan	X	92
26	2013	Mice	Rapamycin	Lifespan/Healthspan	✓	27
93	2014	Mice	GDF11	Healthspan	X	94

Healthspan is commonly interpreted to mean “maintenance of functional health with increasing age”. By necessity, this means one has to understand what it is to be healthy for multiple different systems and tissues. In human beings, this is perhaps non-controversial—one can access high-quality data collected from many thousands of individuals of both sexes as well as differing ethnicities while controlling for multiple lifestyles. One can then establish age-dependent measures for many different aspects of human biology ^35– 41^. These include measures of cardiovascular and cognitive function, movement (walking speed), renal function, and hemodynamic function, to name a few. Typically, such functional measures peak in early adulthood, then decline at different trajectories as the individual ages ^42^. There are many factors that can modulate the slope of such a functional decline with age, including exercise, diet, and lifestyle. Maintaining function and independence with age using selective and specific interventions is arguably the single biggest challenge currently facing geroscience. For the model systems commonly employed in the study of aging biology, identifying functional measures that are relevant to human healthspan is quite difficult. In nearly all model systems used in the biology of aging, healthspan measures have been collected from aging animals not necessarily because of their relevance to human aging but because methods exist that allow one to measure the metric in question over time. Amongst these metrics, there is one clear measure that is very well established as being a robust biomarker of healthspan in human aging, and that is the measurement of movement with age ^43– 45^. A sound argument can be made for measuring this parameter in model systems of aging to ensure potential translational relevance.

## It’s all about the movement!

For some time, it has been known that movement, especially walking speed, is correlated with increased longevity and a reduction in morbidity in human beings ^46, 47^. Movement is perhaps the simplest metric to measure as a functional output of age. Despite its apparent simplicity, walking is a highly complex task, which integrates many different systems including balance, strength, cognitive function, and multiple senses. Walking speed is therefore an integrative physiological outcome, which may be why it has been so tightly linked to the maintenance of health in the elderly. A reasonable extrapolation is therefore to understand the relationship between overall activity and aging pathophysiology. There are large-scale efforts underway to better understand how activity levels modulate longevity, resistance to disease, and function in human beings using personalized tracking devices such as the Fitbit, Apple Watch, or similar devices. Arguably, we should have a deep understanding of how activity levels modulate aging and health in model systems due to our complete control over the environment and genetics. In addition, the economics of carrying out such studies in models are far more practical for obvious reasons.

## Movement as a healthspan metric in model systems

Unfortunately, the literature is hardly replete with such studies. In fact, we are in the infancy of beginning to understand how activity modulates healthspan in model systems. There have been sporadic reports correlating a decline in movement with age for more than 30 years in diverse model systems of aging ^48– 58^. These studies typically use a variety of different approaches to relate movement rates with age or with measures such as gene expression or some other “omic” outcome. There are comparatively few reports in which objective measures of movement rate have been taken, particularly with regard to high-resolution temporal density. Another area not commonly studied is the capture of individual variation with movement and age. Model systems offer the option of outstanding control over the environment, diet, and genetic background. In theory, it would be possible to track individual movement rates in flies, worms, and mice for thousands of individuals, many more than is practical for human beings. Yet, in general, such studies have not been undertaken.

In contrast, most activity in geroscience research using popular model systems has focused on increasing lifespan, with the implicit understanding that if one statistically increases lifespan by even a few percent, then one is by definition working on mechanisms germane to the study of aging ^18^. Increased lifespan is often *de facto* equated with an aging mechanism and is considered the gold standard in geroscience. Yet, for the vast majority of such reports, there is a corresponding lack of knowledge as to whether or not healthspan is increased concomitant with lifespan extension. There are hundreds of publications that identify and characterize genes that “regulate aging”. In contrast, research on defining healthspan (epitomized through studying movement, for example) is relatively unexplored. However, understanding healthspan in these model systems is an absolute prerequisite for beginning to develop pharmacological approaches that extend life in human beings. The reason this is so critical is that increased longevity without increased healthspan is a non-starter. It is unclear whether or not increased lifespan equates to increased healthspan in model systems in general. The prior statement may be considered provocative, as there are many reports in the literature that claim healthspan is increased with lifespan. These studies typically focus on a single gene, which, when mutant, increases lifespan. Such studies, however, typically raise more questions than answers, and these questions need to be robustly addressed before we can unequivocally make the statement that increased healthspan is concordant with increased lifespan for genetic or pharmacological interventions in aging.

It is encouraging that this area of geroscience is beginning to receive more attention. This is exemplified in *C. elegans* with the recent publication of two diametrically opposing articles: one group concluded that increased healthspan of the highly cited longevity mutant *daf-2* results in decreased healthspan (poorer health with longer life) ^59^. Another group argued the exact opposite (maintenance of health with longer life) ^60^. Both studies have merit, but both studies sampled the available biological space of movement over life with low resolution. For example, in the study by Bansal *et al*., movement was assessed for just five minutes every fifth day to determine movement rates over lifespan. This sampling represents roughly 0.07% of the potential biological space in the five-day period. As there was some concordance between replicate measures over time, it was assumed that the measured movement rates were consistent throughout the day and night. No data are provided to support this assumption; however, similar to Bansal *et al*., Hahm *et al*. also carried out fractional sampling of the biological space in their assessment of movement with age. They collected just five seconds of movement data out of every 24 hours (0.006% of the potential biological space) and claimed this as being representative. In addition, the numbers of animals measured in both studies are quite modest, being of the order of a few dozen individuals measured at most, rather than hundreds or thousands that would be typical in human studies. Both of these studies on aging *C. elegans* used more objective approaches to quantitate and track movement, and the research community is rapidly moving away from the more subjective measures of the past ^49, 53, 58^. Both studies also raise a number of intriguing questions with regard to definitively answering whether or not healthspan is increased with lifespan in *C. elegans* (or *Drosophila*, or even mice):

- When measuring movement in a specific time interval, does the amount of movement per time interval change throughout the course of a day/night? What is the impact of circadian rhythms for various genetic or pharmacological interventions?
- How often should one measure movement throughout a lifespan? What is the appropriate measure to determine movement? Many possibilities exist: for example, maximum velocity, total distance moved per unit time, or perhaps a combination of metrics?
- Do movement rates change over lifespan with different diets/laboratory environments? What is the impact of variation between labs?
- Do movement rates over life change between different strains/species? Is there scaling of healthspan relative to lifespan between strains/species?

## Cross-sectional versus longitudinal study design

Many of the questions posed above can be comprehensively answered using automated video capture systems, and appropriate computational infrastructure, coupled with longitudinal analysis. Longitudinal study design is by many considered to be the gold standard in human trials and permits incorporating within-subject variation as well as between-subject variation. Cross-sectional approaches (young to old, for example) largely miss incorporating such variance. Analysis of healthspan in geroscience should be turning to human clinical trials for guidance on experimental design, and longitudinal analysis has many advantages over cross-sectional experimental approaches ^61^.

## Maximizing the advantages of model systems in geroscience research

It seems clear from multiple studies over the last several decades that there is a generalized decline of movement with age in *C. elegans* and *Drosophila.* However, we currently do not have sufficient information to subsample a fraction of the animal’s life for movement and then assume that measure is representative over the entire lifespan. *C. elegans* move with distinct speeds and patterns of movement dependent on the presence of food and their age. It is entirely feasible to thoroughly enumerate this over life. Such data tracking would then allow us to determine how representative a sample of five seconds of movement is for each 24 hours. This kind of rigor should be applied more generally in geroscience experimental design, and the advantages of the experimental system should be exploited, not minimized. Such methodological concerns also apply to genes that have been linked to increased lifespan. For example, if the model organism’s lifespan is increased by 50%, then is it a healthier 50%? Is the lifespan change reflected by increased, sustained, or reduced activity levels? These questions may seem somewhat mundane and not as exciting as mapping pathways or identifying additional genes that modulate lifespan using conventional genetic approaches. However, we currently do not know the answer to most of these “quality of life” questions for many genes or pharmacological interventions, and therefore it makes it very difficult to answer with precision whether or not drug/gene X is improving healthspan. There is a growing effort to acknowledge these issues ^44, 62^ and better define healthspan as something that is standardized. More precise experimental definitions of healthspan will allow us to determine clear and unambiguous outcomes that may be translationally relevant, allowing us to capitalize on the strengths of the invertebrate systems.

## Technology is a moving target in geroscience

Continuing the discussion of movement as a proxy for healthspan, how should one measure movement in invertebrate model systems of aging? Movement of *C. elegans* on the two-dimensional surface of the agar plates on which they are typically housed (with or without food) is conceptually simple to track with age. This can be done in either liquid or solid media, although liquid media is not common in aging studies. Liquid media may have additional concerns as an experimental medium, as *C. elegans* did not evolve in an aquatic environment. There are also newer approaches to measure movement using microfluidic chips ^63– 65^. However, such chips may remain somewhat specialized and may not be widely adopted owing to laboratory-specific expertise. Quantitation of movement in *Drosophila* is more difficult, as adding a third dimension (flight) makes evaluation of the inherent dynamics of movement more problematic. Here too, there have been encouraging efforts using sophisticated cameras/computational approaches to document flight speed and activity with age ^55– 57, 66^. There are also some more “low-tech” approaches to quantitating *Drosophila* healthspan with regard to movement (for example, climbing activity ^67^). Such approaches are somewhat more subjective and may suffer from lab to lab variation with regard to implementation. Tinkerhess *et al.* describe a device in detail for “exercising” *Drosophila*, which may introduce some standardization in this problematic area. However, whether or not such standards become common practice will depend on the degree of adoption by the greater research community. Widespread adoption of a commonly agreed upon method for evaluating movement is critical for replication purposes. Having focused on movement as being the gold standard for healthspan measures in aging invertebrates, there are some alternative measures that have also been employed to assess healthspan, but these tend to be more idiosyncratic and may be model specific, so that the translational relevance to human aging is not clear.

## Other healthspan metrics in invertebrate models

Although a decline in cell number/cell volume for multiple tissues has been documented in aging human beings for several tissues, similar approaches in model systems in aging are not as well established. Adult *C. elegans* comprise 959 cells across multiple tissues, including the musculature, nervous system, pharynx, intestine, reproductive organs, and epidermis. Perhaps the closest parallel of tissue-specific aging in worms compared to humans is the loss of muscle mass with age. Loss of muscle mass is well established in human beings and is termed sarcopenia ^68^. Recently, the van Loon group concluded that the loss of muscle mass with age can be explained by atrophy of type II fibers and the commonly held belief that individual fiber loss with age was erroneous ^69^. What makes this particular study so compelling is that it was done on the same individuals over time, in contrast to previous studies which were largely cross-sectional in nature (i.e. young versus old). The definition of sarcopenia is constantly being re-evaluated and is currently defined not only by loss of muscle mass but also by loss of muscle quality (i.e. weakness) ^70^. Loss of muscle mass in aging worms was first observed by the Driscoll group in 2002 in a seminal paper describing various aspects of the pathobiology of the aging worm ^53^. It was reported that the 95 individual cells comprising body wall muscle were observed to atrophy and fragment with age, visualized through muscle-specific green fluorescent protein (GFP) reporters ^53^. On the surface, it would appear to be difficult to measure muscle quality (strength) in worms, but recent advances using microfluidic technology have enabled force measurements to be evaluated for worms captured in a microfluidic device. Young worms exert ~34 µN of force when thrashing in liquid media and can move specialized posts in a microfluidic device a distance of 20.36 µm ^63^. This type of methodology could be applied to aging worms in conjunction with muscle-specific reporters as in Herndon *et al*. to evaluate not only muscle quantity with age but how well the muscle functions. Arguments for other potentially related measures such as thrashing rate in different density liquids can also be made, but it is far from clear how such measures relate to sarcopenia in mammals.

Muscle is not the only tissue to degenerate in aging worms. We previously evaluated intestinal integrity with age and determined that there was a stochastic degradation as well as a decrease in the absolute number of cells comprising the intestine ^71^. Presumably, this change has functional consequences for the digestion of food in aging animals. However, it is difficult to relate such outcomes to intestinal aging in mammals, as there is no clear homologous pathology in the elderly. We also reported a loss of specific hypodermal nuclei with age in *C. elegans* ^72^, but, again, the implications for the healthspan of the aging worm are not straightforward. One of the more striking features of the pathobiology of the aging worm is a substantial growth of uterine masses with age ^72, 73^. This seems to be a robust phenomenon of nematode aging having been qualitatively described in a previous report ^74^. This germline pathology appears to be modulated by a decline in *cep-1/*p53 with age ^73^. One clear outcome of the increase in uterine masses in the aging nematode is the massive proliferation of DNA copy number per worm. As individual animals age, there is as much as a fivefold increase in genome copy number per worm, directly related to endoreduplication in the gonad. The implications for the health of the animal are again not clear, and it is even less clear if there is a straightforward parallel to healthspan in aging humans.

The widely used long-lived mutant *daf-2(e1370)* has nearly double the genome copy number per individual animal compared to the wild-type ^73^. This is observed even in young animals with the *daf-2(e1370)* allele, despite being somewhat less fertile than wild-type controls and containing less progeny. It is formally possible (but unlikely) that the extra genome copies are due to additional somatic cells indirectly derived from the *daf-2* mutation. Alternatively, perhaps there is endoreduplication of specific cell types. Unfortunately, the origin of these extra genome copies currently remains unknown. More work is needed with regard to genome/cell number in the aging worm. One of the worm’s clear strengths is that it remains almost unique in experimental systems in that a complete understanding of the cell fate map from development to adulthood has been elucidated. It is possible that extra genomes in the *daf-2* mutant allow for an increased reserve capacity against somatic mutations with age and therefore maintenance of tissue homeostasis. Such an explanation has been advocated to explain the resistance of elephants to cancer, as they have 20 copies of the tumor suppressor gene *p53*, as opposed to humans, who have only one. On a cell number basis alone, elephants would be expected to have much more cancer late in life than ourselves, yet they have a cancer incidence of only 4.8% compared to 11–25% in ourselves ^75^, perhaps due to the extra copies of *p53* in the elephant genome allowing for more robust tumor suppression. Similarly, perhaps critical extra genome copies in the *daf-2* background provide a “reserve capacity” buffering life-limiting pathologies in aging worms. Regardless, the increased genome copy number in *daf-2* is at present a curiosity, and the functional consequences remain unexplained.

Other pathological hallmarks that appear to change with age in *C. elegans* include altered neuronal architecture of aging worms ^76, 77^ and an increase in age-related pigments ^78^. There have also been reports of a decline in reproductive fitness with age in *C. elegans* ^79^. Reproductive health is generally not a focus of geroscience, as the elderly face many more serious health problems than their ability to reproduce. For a number of the diverse aging phenotypes reported in *C. elegans*, many seem to arise well before mean lifespan, and the dynamics over life from lab to lab or influence of genetic background are typically not known. In *Drosophila* too, there have been a number of reports of age-related changes in different organ systems such as the intestine and germline ^13^. Again, the functional consequences for healthspan are not clear for reasons similar to those articulated in describing the aging worm intestine. For a tissue-specific decline in organ function with age, the fly has one clear homologue of human organ aging: it has a beating heart with many features in common with the mammalian heart and has been used to investigate invertebrate cardiac aging in a number of studies ^14^. Remarkably, there have even been reports describing the benefits of exercise on the aging fly heart ^67, 80^, and this is an exciting research area which needs to be more broadly studied. Unfortunately, there are only a few labs that have the ability to assess cardiac function in the context of diet, genetic background, or individual variation. Given the plethora of genetic tools and strains available in *Drosophila*, a more widespread investigation of cardiac aging would be very powerful to help address functional changes in the aging *Drosophila* heart. Regardless of the reported association with age of each of these diverse phenotypes, they are often reported in the context of healthspan. However, without understanding the functional consequences for the aging animal with a high degree of precision, it is difficult to relate such measures to homologous outcomes of healthspan in human beings.

## Healthspan measures in aging mice

Functional decline in human beings occurs with increasing age, including a decrease in activity, cognition, bone quality, and other multiple reduced organ or tissue functions. We know that such systems decline from endogenous mechanisms of aging, as the performance of elite athletes of all disciplines declines with age quite markedly. One can make the argument that human physiology is optimally defined in an elite athlete, in which diet, lifestyle, and environment have all been optimized to produce peak performance. Yet, even in these individuals, each functional domain of aging declines with age. However, for mice, much of the data describing similar functions are relatively poorly characterized, relying on data from a few recent studies ^81, 82^ or reports from several decades ago. Data on healthspan in mice generated from the 1990’s and before are particularly difficult to relate to contemporary studies. This is because of animal housing practices being quite different in the past compared to current standards of care. In stark contrast to our understanding of healthspan with age in human beings, we know remarkably little about the impact of diet, housing, and genetic background on functional domains of healthspan in mice. Much work needs to be done to address this deficit before we can begin to reasonably assess whether or not pharmacological interdiction with any intervention in aged mice slows or improves function in specific tissues ^62^. Particularly exciting is the development of new technologies that enable non-invasive surveillance of many critical tissues in live mice. Many of these technologies did not exist prior to the turn of the century, so there are exciting opportunities to define in exquisite detail functional decline in different tissues and systems in multiple genetic backgrounds and species ^83– 85^. For example, amazing advances in cardiovascular surveillance via ultrasound with fantastically high frame rates (>1000 frames/second) are possible, facilitating the study of vessel aging *in vivo* ^86^. Improvements in micro-computed tomography (micro-CT) enable whole body scans in as little as eight seconds with minimal radiation exposure at excellent resolution to allow the study of *in vivo* bone aging ( http://bruker-microct.com/products/1278.htm). Whole-body metabolism and activity can also be studied over time with extremely high data rates (data collected every second for days!) with new advances in metabolic cages ( http://www.sablesys.com/products/promethion-line/promethion-cages/). There are also tremendous advances in the assessment of function in the brain via positron emission tomography/single photon emission CT (PET/SPECT) and magnetic resonance imaging (MRI), with extraordinary detail being revealed through these powerful new imaging technologies. Suffice to say that all these improvements in longitudinal surveillance of aging animals provides enormous opportunity to define in great detail how tissues change in function with age in conjunction with targeted pharmacological interventions.

## Pharmacological intervention for increased healthspan/lifespan

Since the early 2000’s, there has been an increasing focus in the study of aging by manipulating lifespan through pharmacological approaches ^20, 22, 23, 29, 59– 62, 87– 90^. The 1990’s could be argued to be the era of “genes for aging” in geroscience research, and in the second decade of the 21 ^st^ century, there has been an explosion of interest in identifying robust pharmacological interventions for lifespan. Healthspan effects have been a secondary consideration until now, but this too is changing with increasing reports of late-life interventions in aging mice to increase lifespan, coupled with healthspan studies ^28, 91– 96^. The intervention testing program administered by the National Institute on Aging (NIA) has been an invaluable advocate in developing this concept ^97^. Initially formulated in the early 2000’s as a multi-center testing vehicle for “pro-longevity” agents, it has popularized the experimental design of a multi-site trial for intervening in aging. The Intervention Testing Program (ITP) consists of three geographically distinct sites (University of Michigan, University of Texas Health Sciences Center, and Jackson Labs), each of which independently evaluates the efficacy of specific pharmacological interventions for extending lifespan in a single strain of genetically diverse mice. The goal of the ITP is to robustly identify interventions that extend life, and although interventions are tested from young adults in some cases, the main goal is to identify late-life interventions. This approach is especially relevant when one considers translational impact, as it is difficult to imagine prescribing a pro-longevity intervention to young adult humans. Far more realistic are targeted efforts in the elderly population. More recently, the ITP has begun to transition from evaluating lifespan alone to assessing select functional outcomes. This is a welcome development, although functional outcomes need to be carefully characterized in the context of human aging if the maximal impact is to be realized. Detailed investigations into the variance of aging phenotypes in untreated animals with functional consequence are a necessary pre-requisite in the effort to precisely understand the impact of any potential pharmacological interdiction.

The overall ITP approach has also given birth to the *Caenorhabditis* ITP (CITP) program. The goals of the CITP are very similar to that of the ITP, but it focuses on identifying robust chemical responses across distinct genetic backgrounds by utilizing genetically diverse species and strains of nematodes. The CITP too has three geographical testing sites for the purposes of replication: the Buck Institute for Research on Aging, Rutgers University, and the University of Oregon. The CITP program is attempting to standardize many aspects of geroscience (survival, lifespan extension, etc.) in the aging worm and to assess healthspan as well. One can see a future in which interventions are evaluated in the CITP program and chemical “hits” that robustly affect lifespan at all three sites are then evaluated for healthspan (movement is perhaps the low-hanging fruit here). Such hits would then subsequently be prioritized for testing in the ITP. The ITP today has evaluated at least 25 interventions in mice and has an approximately 10% hit rate in terms of statistically significantly increasing lifespan. It is beyond the scope of this article to discuss in detail the many pharmacological approaches reported for intervening in aging. However, it is worth discussing two highly visible examples in this area.

If interventions that are robustly positive for lifespan extension are also positive for healthspan extension, then we have a very powerful system for the prioritization of preclinical interventions for aging in human beings. Arguably, rapamycin is the first robust outcome from the ITP in this regard, with multiple reports of lifespan extension in mice and some reports of healthspan extension as well ( Table 1). We previously reported that cardiac health in elderly female mice was improved by a short rapamycin treatment late in life ^26^. This was later confirmed in similar experiments by another group ^27^. However, in another investigation on late-life rapamycin treatment in males only, no significant benefits were reported ^25^. In addition, there are clear deleterious effects from chronic rapamycin treatment in mice. Negative outcomes include testicular atrophy and increased incidence of cataracts ^28^. Clearly, more work needs to be done to address the potential for sex-specific responses to rapamycin with regard to healthspan effects as well as adverse consequences resulting from pharmacologically attenuating aging. We are clearly in the beginning of developing and characterizing robust interventions in preclinical models for aging, but where are we in human trials?

Preliminary trials in human beings to reduce morbidity and extend healthspan/lifespan are either in process or in the planning stage at multiple sites around the world. These efforts are in part capitalizing on the outcomes from geroscience in model organisms over the last three decades. One example is the TAME (Targeting Aging with MEtformin) trial, recently discussed in the popular press and literature ^98^. This trial is built in part on successful studies in aging model systems treated with metformin ^20, 99, 100^ as well as data from a recent meta-analysis of diabetics. A significant motivating factor in this trial is the excellent safety profile of metformin, which has been in use for nearly 60 years. The approach is to determine whether chronic metformin treatment in the elderly improves health and reduces co-morbidity for multiple indications. Other work recently completed in this context is a limited trial with the mTOR inhibitor RAD001—a molecule similar to rapamycin that also decreases mTOR activity ^101^. This trial focused on a vaccine response in the elderly: older individuals were pre-treated with RAD001, which, perhaps counterintuitive to conventional wisdom, resulted in an improved immune response to an influenza vaccination compared to an untreated control group. This is consistent with a variety of model systems in a geroscience context in that down regulation of mTOR appears to benefit function for many systems (including the immune system) in aged animals. One commonality in both candidate interventions is the fact that the interventions were already FDA approved and have known safety profiles. This type of approach is likely to be the most straightforward way to aggressively move into trials for intervening in aging, as the length of time required to develop novel pharmacological interventions will require many years and is subject to stringent approvals at multiple levels.

Regardless of the initial success or failure of initial candidate molecules in the human arena, it is quite likely that the pace of such work will increase in the near future owing to growing demand for biomedical solutions to increasing healthcare costs as the baby boomer generation continues to age. The conserved biology of aging coupled with multiple successes in extending lifespan/healthspan in geroscience research on model organisms give a great deal of hope that we will identify effective and precise therapeutics to combat the functional decline of aging and perhaps increase lifespan as well.
